# Biofilm tolerance, resistance and infections increasing threat of public health

**DOI:** 10.15698/mic2023.11.807

**Published:** 2023-09-26

**Authors:** Shanshan Yang, Xinfei Li, Weihe Cang, Delun Mu, Shuaiqi Ji, Yuejia An, Rina Wu, Junrui Wu

**Affiliations:** 1 College of Food Science, Shenyang Agricultural University, Shenyang 110866, P.R. China.; 2 Liaoning Engineering Research Center of Food Fermentation Technology, Shenyang 110866, P.R. China.; 3 Shenyang Key Laboratory of Microbial Fermentation Technology Innovation, Shenyang 110866, P.R. China.

**Keywords:** biofilm, tolerance, resistance, diseases, intervention

## Abstract

Microbial biofilms can cause chronic infection. In the clinical setting, the biofilm-related infections usually persist and reoccur; the main reason is the increased antibiotic resistance of biofilms. Traditional antibiotic therapy is not effective and might increase the threat of antibiotic resistance to public health. Therefore, it is urgent to study the tolerance and resistance mechanism of biofilms to antibiotics and find effective therapies for biofilm-related infections. The tolerance mechanism and host reaction of biofilm to antibiotics are reviewed, and bacterial biofilm related diseases formed by human pathogens are discussed thoroughly. The review also explored the role of biofilms in the development of bacterial resistance mechanisms and proposed therapeutic intervention strategies for biofilm related diseases.

## INTRODUCTION

Biofilm is a ubiquitous form adopted by microorganisms in nature. Microbial cell forms aggregates or clusters and is embedded in its self-generated extracellular polymeric substance (EPS) [1]. In contrast to free-living planktonic microorganisms, biofilms form a physical scaffold with three-dimensional structure to maintain the organizational structure of microbial communities, which further allows communication and synergy between specific species [2]. Such tissue structure makes the microorganisms in the biofilm protected from external interference and increases the resistance to mechanical external forces and antibiotics [3]. In addition, the antibiotic resistance of microorganisms may also be regulated after the formation of biofilm [4].

Human beings coexist with microorganisms, which have an important impact on the physiology and health of the host. Some microorganisms symbiotic with the host aggregate into biofilms, for example, on the intestinal, vaginal or oral mucosas, and skin [5–7]. However, the colonization of hosts by pathogenic microorganisms and the formation of pathogenic biofilms can lead to recurrence and chronic infection (biofilm infection) [8]. Biofilm is problematic because they have tolerance and drug resistance and can escape human defense mechanisms, thus hindering the treatment of infection [9].

Both tolerance and resistance are related to the resistance of biofilm to antibiotic treatment [10]. In terms of mechanism, drug resistance is caused by acquired mutations, which usually involve antibiotic degrading enzymes, target mutations or efflux pumps. These mutations eliminate the molecular targets of antibiotics and enable bacteria to have antibiotic resistance even if they are not encapsulated in biofilm. However, antibiotic resistant cells in biofilms can survive under high concentrations of antibiotics only if they are encapsulated in biofilms. The term antibiotic tolerance can also be used for planktonic bacterial populations. Here, it describes the survival of bacterial cells in the presence of bactericidal antibiotics without acquiring the genetic determinants. Antibiotic tolerance of planktonic bacteria is mainly caused by the changes of cell physiological state caused by environmental stress and mediated by cell stress response and related systems [11]. In the biofilm, whether attached to the surface or not, bacteria gather in the endogenous extracellular matrix to form a structured environment different from planktonic cells. The structured environment of biofilm leads to the development of tolerant subsets of cells, and the tolerance mechanism of biofilm is mostly related to these tolerant subsets, for example, with extracellular matrix or anaerobic conditions, which is different from planktonic cells [12, 13]. The tolerance of microbial biofilms to components of the host immune system and antibiotics is the main cause of their infection [14]. The current prevention and treatment measures are mainly antibiotics. The use of antibiotics may reduce the number of bacteria in the biofilm, but they cannot completely eradicate the biofilm, so the recurrence of biofilm infection often occurs [15].

Exploring the tolerance and resistance mechanism of biofilm will help us to develop effective methods to treat persistent infection. In this review, we first describe the composition and characteristics of biofilms. Subsequently, we discussed the tolerance mechanism of biofilm to the immune system and the understanding of antimicrobial tolerance and drug resistance. Then, different types of biofilm infection and related clinical problems were discussed. Finally, the urgent problems and future research strategies are put forward to face the challenge of treating biofilm infection.

## BIOFILM FORMATION AND CHARACTERISTICS

**Figure 1:**
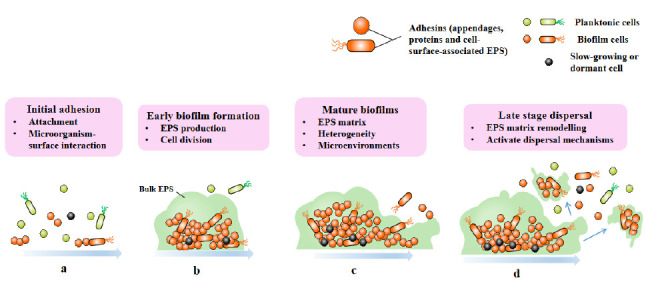
FIGURE 1: Four stages of biofilm formation. Biofilm-formation can be divided into four stages: attachment (a), proliferation (b), maturation (c), and dispersion (d).

Biofilms are three-dimensional microbial communities that adhere to biological or non-biological surfaces and are encapsulated by EPS, extracellular DNA (eDNA) and extracellular proteins secreted by cells [16]. The formation of biofilm is a development process that includes four different stages: attachment stage, proliferation stage, maturation stage and dispersion stage (**Fig. 1**) [17]. Planktonic bacteria attach to abiotic and biological surfaces through physical forces such as spatial interaction, Van der Waals forces, electrostatic interaction and bacterial appendages such as fimbria and flagella [18]. Adhesion refers to the attachment between bacteria and surfaces, and the connection between cells and bacteria is called cohesion [19]. Bacterial cells begin to communicate with each other by forming self-inducing factors that lead to the expression of specific genes related to biofilm [20, 21], and stabilize biofilm by producing EPS matrix [19]. In addition to EPS, eDNA is also involved in biofilm stabilization and bacterial communication [22]. Then comes the proliferation and maturation stage, including the production of extracellular matrix and the development of three-dimensional biofilm structure. The last step is dispersion, and the bacterial biofilm forms various enzymes, which will reduce the stability of extracellular polysaccharides, so that the bacterial cells located on the surface of the biofilm are released and colonize a new surface, leading to the spread of infection [19].

The transition from plankton to biofilm involves a series of physiological, metabolic and phenotypic changes, which are coordinated by the secondary messenger cyclic diguanoside-5'- monophosphate (c-di-GMP), as exemplified by *Pseudomonas aeruginosa* [23]. High levels of c-di-GMP induce biofilm formation, while low levels of c-di-GMP lead to biofilm dispersion [24, 25]. With the cell division of biofilm, the composition complexity of matrix and number increase, forming a microenvironment characterized by nutrients and oxygen gradients in biofilm. This makes cells in biofilm having physiological heterogeneity and adaptability, and this differentiation is constantly changing and strictly regulated [8].

## OTHER PROTOZOAN PARASITES OF MEDICAL RELEVANCE

### Pathogen associated molecular patterns (PAMPs)

After infection, the innate immune system will make a non-specific initial response to attack the invading microorganisms [26, 27]. Lipopolysaccharides (LPS) from Gram-negative bacteria directly activate the complement system, thereby attracting polymorphonuclear leukocytes (PMNs). Resident macrophages can recognize invading microbial pathogens and recruit PMN. Pathogen recognition receptors (PRRS) (receptors on PMN and macrophages) have the ability to mediate the recognition of invading microorganisms by the innate immune system. The production of innate immune response is caused by the combination of PRRS and conservative pathogen related molecular patterns (PAMPs).

### Biofilm related molecular patterns (BAMPs)

For a long time, there has been no report of biofilm-specific PAMPs. However, recent studies have shown that molecular patterns exist in both biofilm and planktonic bacteria, and will lead to innate immune response, and when expressed in biofilm, the innate immune response is stronger [8]. This PAMPs subgroup was recently found in the biofilm matrix of *P. aeruginosa* and is known as “biofilm related molecular patterns” (BAMPs) [28]. The immune stimulation characteristics of biofilm may be related to BAMPs, and the degree of degranulation and respiratory burst in response to PMN may be determined by the specific extracellular polysaccharide composition of *P. aeruginosa* biofilm [29]. BAMPs include the matrix exopolysaccharides alginate, Pel (pellicle) and Psl (polysaccharide synthesis locus), filamentous Pf bacteriophages and LPS [30].

### Tolerance to the immune system

Even if the innate immune response has been activated, the establishment of biofilms may enable bacteria to proliferate. When the host response is further enhanced by activating the adaptive immune system, the biofilm can even protect the embedded bacteria. The adaptive immune system involves the maturation and release of IgG and proinflammatory cytokines, which then leads to collateral tissue damage [31].

EPSs cause immune evasion in two ways, one is by shielding PAMPs on the surface of bacteria, and the other is by its mechanical protection [32]. Although the host's immune response mechanism may reduce the number of bacteria, surviving persistent cells may regenerate and lead to the recurrence of disease symptoms [33]. The formation of biofilms increases the resistance of bacteria to human defense mechanisms and antibacterial treatments, thereby promoting chronic infections. Biofilm can also serve as an environment to accumulate different types and quantities of bacteria at certain locations.

## TOLERANCE OF BIOFILM TO ANTIMICROBIAL AGENTS

### Heterogeneity of biofilm

Biofilm constitutes a subgroup of rapidly growing, metabolically active cells located at the gas-liquid interface, while the slow growing, metabolically inactive, or non-growing cell subgroups in the deep layer of biofilm, which corresponds to the spatial distribution of oxygen and nutrients [34]. Moller *et al.* used the continuous in flow cells system, which can monitor the development process of biofilm in real time and non-destructively [35]. In flowing cells, bacteria grow on the glass surface with the continuous influx of oxygen-containing growth medium. The thickness and biomass of biofilm will increase over time, leading to the formation of metabolites and oxygen gradients, resulting in physiological heterogeneity. The result is a layered biofilm with growth rate, metabolic activity, antibiotic tolerance and internal gradient of oxygen [36].

### Interaction between antibiotics and biofilm matrix

**Figure 2:**
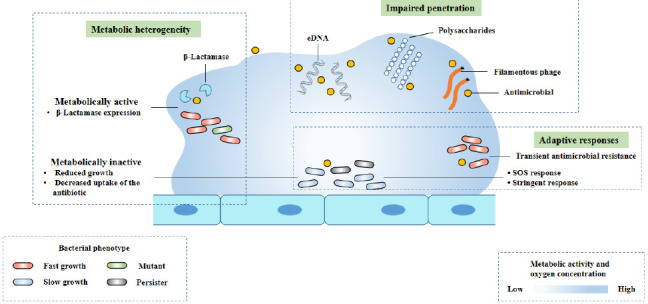
FIGURE 2: The mechanisms of antimicrobial tolerance of a biofilm. Modified from Ciofu [8]. This diagram shows three known mechanisms of antimicrobial drug tolerance: metabolic heterogeneity, impaired permeability, and adaptive responses to stress and antibiotics.

The interaction between biofilm matrix and antibiotics (**Fig. 2**) is determined by the physical properties of antibiotic molecules (such as molecular size and charge on the surface of antibiotic molecules) and the matrix composition of biofilm. Cationic antibiotics can react with the matrix components of biofilm, such as eDNA and polysaccharides, which have an overall anionic charge. This combination causes a decrease in the diffusion rate of antibiotic molecules in the biofilm [37–40]. Due to the slow diffusion rate, biofilm cells have enough time to activate adaptive stress response, which contributes to the enhancement of tolerance [41]. In Gram-positive and Gram-negative bacteria, some antibiotic modifying enzymes come from the matrix, such as β-Lactamase, which are able to inactivate antibiotics before they reach bacterial cells, and can enrich in the outer layer of biofilm (**Fig. 2**) [42–44]. Filamentous phages may also help slow the diffusion of antibiotics through *P. aeruginosa* biofilm (**Fig. 2**). Studies have shown that Pf phage plays a role in matrix assembly and biofilm diffusion. In addition, some researchers believe that the production of Pf phage can induce the release of eDNA and cell lysis in biofilm, and speculate that this may be a regulated process. In the biofilm of *P. aeruginosa*, filamentous Pf phage can enhance the viscosity of self-assembled liquid structure formed by matrix extracellular polymer. Pf phages carry negative charges, which contribute to the binding, tolerance and isolation of antimicrobial peptides and cationic aminoglycosides [30].

Biofilm is a structure composed of microorganisms and a matrix different from surrounding tissues, and should be considered as a third pharmacokinetic chamber, distinct from blood (first chamber) and interstitial fluid (second chamber), as they exhibit different antibiotic pharmacokinetics from surrounding tissues. Antibiotics used systematically are difficult to reach this independent compartment [37, 38, 45].

### Trigger adaptive stress responses

As mentioned earlier, biofilms are bacteria whose nutrient and oxygen gradients represent spatial tissue stress conditions, which in turn triggers adaptive responses such as induced SOS response, general RpoS response and stringent response, impairing the efficacy of antibiotics and leading to antibiotic tolerance of biofilms (**Fig. 2**).

The stringent response of bacteria is a widely conserved adaptive stress response to iron starvation and nutrition, which changes cell physiology by activating the production of guanosine 3',5' - bispyrophophate (ppGpp) [46]. In biofilm, stringent response regulates and down regulates physiological factors in a bacterial subgroup with weak metabolic activity. By preventing the accumulation of active oxidizing substances and activating stringent response, *P. aeruginosa* biofilm can be tolerant to fluoroquinolone, meropenem and gentamicin, Nguyen et al. believe that this is a usual mechanism by which antibiotics act on bacteria [47].

SOS response is a stress response to DNA lesion. Researchers have shown that this response promotes the tolerance to fluoroquinolones by inducing the expression of DNA repair mechanism in *P. aeruginosa* and *Escherichia coli* [48,49]. SOS response can also be achieved by external exposure to low levels of antibiotics such as fluoroquinolones, aminoglycosides or β-lactams because they have the same mechanism of action involving the production of ROS. When ROS level is too low to kill bacteria, it will lead to DNA oxidative damage (mutation), and then activate SOS response, repair DNA lesion and obtain antibiotic tolerance [50].

The stress response mediated by RpoS during quiescence is also triggered in the biofilm. The tolerance to β-lactams, such as ciprofloxacin and carbapenems [48], has been shown to be RpoS dependent, indicating a reduced tolerance of biofilms with RpoS mutations to these antibiotics.

In conclusion, the tolerance of biofilm infection to antibiotics is variable and affected by many factors (**Fig. 2**). The relative contribution of different tolerance mechanisms in different types of bacterial biofilm infections are varies. This depends on antibiotic treatment and the type and location of bacterial biofilm infection [39].

## ANTIMICROBIAL RESISTANCE OF BIOFILM

Compared with tolerance, antimicrobial resistance is not transient. Even if the biofilm is destroyed, it will still exist in bacteria. It is caused by bacterial genome mutation or antimicrobial resistance factors obtained through horizontal gene transfer (HGT).

### Mutagenesis in biofilms

Several conditions encountered in the biofilm have promoted the development of antibiotic resistance: the existence of tolerance and persistence of the slowly growing population, the large amounts of bacterial cells in antibiotic treatment, and the antimicrobial selection pressure and local competition in the regional structure of the biofilm among mutants. In non-growing, nutrient deficient bacterial populations, the activation of adaptive stress response (oxidative stress, SOS, RpoS or stringent responses) leads to adaptive mutations [51–53], which, along with mutations in the rapidly growing population located in the outermost layer of biofilm, promote an increase in mutations in the biofilm and a higher mutation rate in the outer layer of the biofilm. Activation of TLS (translational synthesis) DNA polymerases (such as polymerase IV and polymerase III) is a common mechanism for increased mutations caused by stress response. Because they bypass non-coding lesions and do not have proofreading activities, they are inherently prone to errors. Overexpression of TLS polymerase coding gene induced by stress response can increase mutation [50].

**Figure 3:**
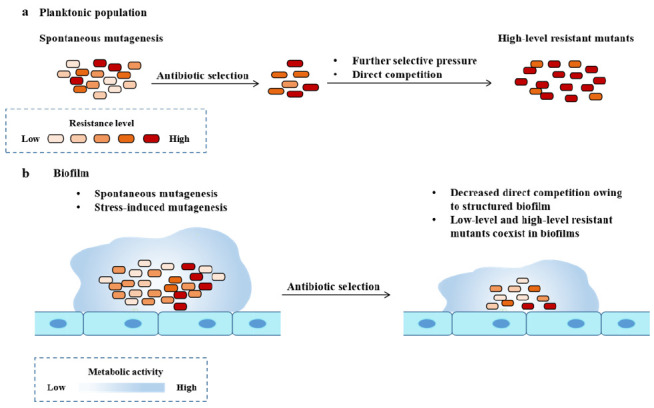
FIGURE 3: Development of antimicrobial resistance in planktonic bacteria and biofilm bacteria. Modified from Ciofu [8].

Compared with homogeneous planktonic bacterial populations, the heterogeneous environment and spatial structure of biofilm lead to different niches with local selection pressure [54, 55], which provides an opportunity for more kinds of resistant mutants to coexist and persist in biofilm (**Fig. 3**) [56, 57].

### Evolution of AMR in biofilms

The evolution experiment showed that compared with the culture of *P. aeruginosa*, the evolution of AMR in biofilm was faster [58]. Compared with planktonic culture, the formation of biofilms promotes the development of mutants with low levels of resistance to quinolones, and its related adaptation cost is low, which is mainly due to the up regulation of efflux pump [59, 60]. Although the bacteria released by the biofilm showed higher MIC than the ancestral strains, the biofilm bacteria may not reach the clinical drug resistance breakpoint used in the routine antibiotic planktonic sensitivity test [61], but the generation of low resistant mutations supports the *in vivo* resistance of the biofilm to antibiotics, and additional resistant mutations can lead to high resistance (**Fig. 3**).

Compared with planktonic bacteria, the differential evolution of mutational resistance in biofilms has been found in other bacteria, including *Acinetobacter baumannii, Salmonella enterica* and *P. aeruginosa*, exposed to different types of antibiotics [62–63]. Parallel evolution across species (*A. baumannii* and *P. aeruginosa*) has been observed in terms of resistance to ciprofloxacin and tobramycin [62]. Nevertheless, the dynamics and type of the mechanisms of resistance in biofilms depend on the selection pressure exerted by different antibiotics with different modes of action and the adaptation cost caused by specific mutations [63]. Although resistance mutations in planktonic culture and biofilm may occur in the same gene, the mutation types between the two bacterial growth patterns and the generation and maintenance dynamics of resistance mutants are different, which distinguishes the evolution of resistance in planktonic culture and biofilm. The tolerance to antibiotics of biofilms and their structural heterogeneity are possible reasons for the specific evolutionary pathway of biofilms [57].

### Horizontal gene transfer (HGT) and gene exchange in biofilms

HGT and gene exchange in biofilm drive the transmission of AMR genes. Because the movement of cells embedded in the matrix is limited, the distance between cells in the aggregate is close, and eDNA in the matrix may produce intercellular contact and provide genetic material for transformation, HGT may be more frequent in biofilms. Conjugation requires close contact between donors and receptors, which may be the most common HGT mechanism in biofilms, particularly in multi species biofilms [64]. In the spatial tissue habitat of biofilm, conjugate transfer is limited to subspecies, small island donors and receptors, which produce transconjugates, but will not trigger the ‘wave' of HGT through biofilm. However, the growth of biofilms leads to the persistence of plasmids, thus accelerating the spread of resistance between pathogens [65].

High binding rate has been demonstrated in biofilm [64], and it is reported that the transfer rate of binding protein in *Staphylococcus aureus* biofilm is 16,000 times higher than that in planktonic culture [66]. The gene exchange of chimeric *pbp* gene (encoding penicillin-binding protein) between commensal *Streptococcus* spp. (*Streptococcus mitis* and *Streptococcus oralis*) and *Streptococcus pneumoniae* in oral biofilm indicates the *in vivo* correlation of HGT in biofilm as the transmission mechanism of resistance [67]. Besides, relevant studies have demonstrated that natural binding can promote the formation of biofilm [68]. The stringent response activated in the biofilm has been shown to play a part in the upregulation of integrase in the biofilm [69]. The study also shows that *P. aeruginosa* in the biofilm can naturally transform genome and plasmid DNA [70], which indicates that HGT may also occur in the biofilm through this mechanism. In addition, antibiotic exposure may increase the incidence of HGT by activating SOS stress response, which may stimulate the transmission of resistance genes [71].

Membrane vesicles accumulate in biofilm matrix, which can transfer antimicrobial resistance genes (ARGs) from chromosome source or plasmid between bacteria and promote HGT [72]. Besides that, the membrane capsule can indirectly support HGT by transporting Quorum-sensing (QS) factors in Gram-negative bacteria, because QS participates in coupling, transformation and phage activation [73].

In biofilm infection, it is difficult to track the transmission direction of ARG, but epidemiological and experimental data show that HGT plays an important role in the transmission of resistance *in vivo* [71]. Above all, HGT mechanism plays a significant role in the development and possible transmission of AMR in biofilms [73].

**Table 1.** Diseases related to microbial biofilms, triggers and common bacterial pathogens.

**Affected organ system**	**Predisposition**	**Common pathogens**
**Skin and soft tissue**		
Pressure ulcer	Long term skin compression	*Staphylococcus aureus* *Pseudomonas aeruginosa*
Foreign-body located chronic wounds	Soft tissue implants	*Staphylococcus spp.* *Streptococcus spp.* *Enterobacteriaceae* *Enterococcus spp.* *Pseudomonas spp.* *Acinetobacter spp.*
**Musculoskeletal system**		
Osteomyelitis	Bone injury sequelae	*S. aureus*
Cardiovascular system		
Endocarditis	Injured endothelium, previous infective endocarditis	*S. aureus* *Streptococcus*
Blood stream infections	Blood stream access device (for example, central venous catheters)	Coagulase negative *Staphylococci* *S. aureus* *P. aeruginosa*
**Respiratory system**		
Pharyngitis and laryngitis	Allergies, immunodeficiency	*S. aureus*, *H. influenzae*, *C. albicans*, *Moraxella nonliquefaciens, Propionibacterium acnes, Neisseria meningitidis*, *S. pneumoniae*
Cystic fibrosis	Primary ciliary dyskinesia, chronic obstructive lung disease, bronchiectasis	*H. influenza* *S. aureus* *P. aeruginosa*
Patients with chronic obstructive pulmonary disease	Air pollution, dust, genetics	*Pseudomonas, Klebsiella, Acinetobacter, Enterobacter, Moraxella catarrhalis and mixed infections.*
**Digestive system**		
Dental caries and periodontal diseases	Poor dental hygiene	Genera *Actinomyces, Lactobacillus, Dialister, Eubacterium, Olsenella, Bififidobacterium, Atopobium, Propionibacterium, Scardovia, Abiotrophia, Selenomonas*
Peptic ulcer disease, esophageal, adenocarcinomas	Fecal oral route and oral route of transmission	*Helicobacter pylori*
Inflammatory bowel disease	Environmental, genetic, infectious and immune factors	*Fusobacterium spp., Shigella spp.,* adhesive *E. coli*
**Urogenital system**		
Acute cystitis	Urinary tract catheterization	Uropathogenic *E. coli* (UPEC) *S. aureus*
Chronic bacterial prostatitis	Disorders of micturition, urolithiasis	*E. faecalis, Staphylococcus spp., E. coli*
**Central nervous system**		
Meningitis	Cerebrospinal shunts	*H. influenzae*
Sensory organs		
Otitis externa	Bacterial infection, foreign body implantation (hearing aid, earphone)	*Streptococcus, Staphylococcus aureus, Pseudomonas aeruginosa*
Foreign body-related keratitis	Contact lenses	*P. aeruginosa*

## RELATIONSHIP BETWEEN BIOFILM AND HUMAN HEALTH AND DISEASE

**Figure 4:**
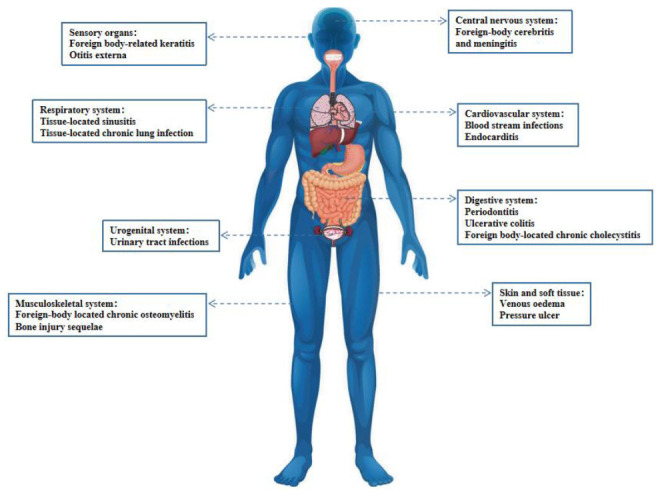
FIGURE 4: Diseases associated with bacterial biofilms.

A National Institutes of Health (NIH) study showed that 60-80% of microbial infections were related to biofilm formation (**Tab. 1**) [74]. Biofilm formation occurs not only on medical appliances, such as catheters, pacemakers, heart valves and prostheses, but also on various body surfaces, including the mucous membrane or skin surfaces of digestive tract and respiratory tract (**Fig. 4**). Besides that, the biofilm formed in the environment is not only potential habitats for pathogens outside the host, but may also trigger new infections. Some studies have shown that the associated bacteria in the biofilm show higher tolerance and drug resistance to antimicrobial compounds than plankton alone [75].

### Medical device

In clinical treatment, bacterial biofilms can form on foreign body implants, for example, orthopedic inserts, catheters (urinary and intravascular), breast and dental implants, which can lead to serious infection [76]. Biofilm on medical equipment has high resistance to antibiotics, which poses a great danger. It provides a bacterial reservoir that can lead to persistent chronic inflammation and reinfection, as well as equipment blockage, tissue damage and resistance to treatment. Important microorganisms related to infection include Gram-positive bacteria, such as *Staphylococcus epidermidis, Enterococcus faecalis*, and *S. aureus* as well as Gram-negative bacteria, like *Klebsiella pneumoniae, E. coli* and *P. aeruginosa* [77].

### The respiratory tract

The respiratory tract is composed of large mucosal surfaces and thus becomes a preferred niche for biofilm growth, which leads to decreased lung function and chronic inflammation of mucosal tissues. For example, chronic sinusitis, a widespread inflammatory disease, may be related to the formation of bacterial biofilms in the upper airway. *S. aureus* biofilm was observed on the surface of nasal mucosa in nearly half of sick patients [78], but other pathogens include *Haemophilus influenzae, Moraxella catarrhalis* and *S. pneumoniae* [79]. The last two are inclined to form biofilms between species, making the treatment more complex.

Chronic phenotypes of laryngitis and pharyngitis are often related to the presence of biofilms. A related study showed that biofilms have been found in over 60% of patients with chronic laryngitis, including *Candida albicans, S. aureus, H. influenzae, Moraxella nonliquefaciens, Propionibacterium acnes, S. pneumoniae* and *Neisseria meningitides* [80]. The formation of a large number of biofilms may explain the necessity of antibiotics in the treatment of some chronic laryngitis.

Chronic infections of the lower respiratory tract are often associated with bacterial biofilms, mainly in susceptible patients with abnormal mucociliary clearance and other impaired host defense, for instance, cystic fibrosis (CF). Chronic lung infection can aggravate the primary disease and lead to destructive inflammation. Changes in the viscosity and transparency of the patient's mucosa may promote the formation of biofilm [81].

Patients with chronic obstructive pulmonary disease (COPD) have a higher risk of acute exacerbation caused by *Pseudomonas, Acinetobacter, Enterobacter, Pseudomonas, Klebsiella*, and other mixed infections [82].

With the increase of these species, the production of biofilms also increased, and this is usually related to clinical isolates. For instance, 85.6% of clinical isolates of *K. pneumoniae* exhibit the ability to produce biofilms; this is also related to multiple resistance [83]. Although the formation of biofilm is always described in the setting of COPD lung infection, most of them lack direct proof of lung biofilm formation, and the verification is still mainly through indirect means.

### Digestive tract

A large number of diverse microorganisms exist in the digestive tract of the human body, most of which are in the colon. Over 700 different bacteria have been found in the oral cavity of humans [84]. They form biofilms on the teeth, also known as dental plaque. Biofilm and continuous inflammation will gradually lead to gingival atrophy, dissolution of periodontal fibers, and bone destruction, leading to tooth loosening and ultimately tooth detachment [85]. Compared with gingivitis, the tissue damage of periodontitis is unrecoverable. Sub gingival biofilms are mainly a variety of gram-negative rods, such as *Prevotella* and *Clostridium nucleatum*, but also include deep moving bacteria and spirochetes close to the epithelial surface [86].

It is worth noting that biofilm plaques are persistent reservoirs of microorganisms and their inflammatory effectors, both of which can spread *in vivo*. Therefore, oral biofilm bacteria are also directly or indirectly related to other systemic diseases, like cardiovascular disease, diabetes, premature birth and low birth weight infants [87].

*Helicobacter pylori* colonization is associated with peptic ulcer disease, major gastritis, esophageal cancer and adenocarcinoma [88]. Urease is one of the virulence factors of *H. pylori* [89]. It is worth noting that 97.3% of the gastric mucosal surface is covered by bacterial biofilm in urease positive biopsies of patients with peptic ulcers, while the average percentage of total surface area covered by biofilm in urease negative biopsies is 1.64% [90]. A recent study also emphasized the importance of *H. pylori* forming biofilm *in vivo*. This study showed that the combination treatment of antibiotics and biofilm destroying compound N-acetylcysteine eradicated *H. pylori* in 67% of patients, while single antibiotic treatment cleared the infection in only 20% of patients [91].

A large number of diverse bacterial microbiota are colonized on the intestinal mucosa and usually grow as a healthy biofilm community [92]. Although clear etiology can lead to different acute diarrhea diseases, the etiology of colorectal cancer, irritable bowel syndrome and inflammatory bowel disease (IBD) and its relationship with clear bacterial species are unclear. However, it is generally believed that intestinal flora has beneficial and adverse effects on these diseases [93, 94]. For instance, ulcerative colitis, a chronic recurrent form of IBD, is associated with multiple biofilms forming species, such as *Clostridium* and *Shigella*. While adherent *E. coli* is related to promoting the initiation and development of disease [95]. In the same measure, Crohn's disease is related to the increase of *Pseudomonas, Bacteroidetes* and *Enterobacteriaceae*. These known bacterial communities can form biofilms. [96].

Bacterial biofilms can facilitate the chronic colonization of bacterial populations in the gut. In addition, the relatively high antibiotic resistance of biofilms is one of the reasons for the difficulty of antibiotic treatment of IBD. Moreover, biofilm matrix components may enhance the pro-inflammatory response, which is a marker of IBD. Some reports have pointed out the significance of bacterial biofilm in the pathogenesis of Crohn's disease and ulcerative colitis, but we currently lack a comprehensive understanding of its mechanism [97].

### Skin and wounds

More than 50% of the microbial load on the skin is to be made up of various bacteria that can form biofilms, mainly including *Propionibacterium spp., Corynebacterium spp*., and *Staphylococcus spp* [98]. These bacteria can cause various skin diseases, such as cellulitis, pustules, necrotizing fasciitis caused by *Staphylococcus pyogenes, Staphylococcal* graded skin syndrome caused by *S. aureus*, and chronic wounds caused by otitis externa and *P. aeruginosa*. Generally speaking, biofilms increase the adaptability of bacteria to host immune defense, antibiotic treatment and general health treatment. Bacterial biofilms can also affect infection and chronic wound healing, as they are related to wound development and increased skin infection and improper wound healing caused by chronic inflammation [7].

Related studies have confirmed that the skin tissue of chronic wounds contains a variety of bacteria that can form biofilm, such as *S. aureus, S. epidermidis, K. pneumoniae*, and *E. faecalis*. *S. aureus* was detected in nearly 88-98% of wound infections [99]. *S. aureus* has fibrin receptor, so it can bind to fibrinogen and start the formation of biofilm. Infected patients need to prolong healing time because re-epithelization of infected tissue delays healing. *S. aureus* biofilms are difficult to tolerate antibiotic treatment and host immune response [100]. The presence of cytokines and β-lactam antibiotics even promotes the production of biofilm [101]. Chronic wounds do not always contain a single strain of chronic infection and can coexist with some kinds of different biofilm producing strains, like *P. aeruginosa* and *S. aureus*. Recent data suggest that these two bacteria benefit from each other in coinfected wounds and produce synergistic effects to increase antibiotic tolerance [102].

The new data also show that the formation of biofilm is a key pathogenic factor of opportunistic pathogen acne, which is related to the inflammatory disease acne vulgaris and soft tissue, skin, cardiovascular system and implant related infections [103]. The formation of biofilms in sebaceous follicles may lead to the increase of drug resistance of *P. acnes* [104]. Compared with the healthy control group, biofilm like aggregates of acne is more common in skin biopsies of patients with acne vulgaris. In addition, the latest data indicate that biofilm formation of *P. acnes* is phylotype-dependent, and compared to healthy skin isolates, isolates from invasive infections have stronger biofilm production ability [105, 106].

## THERAPEUTIC INTERVENTION STRATEGIES FOR BACTERIAL BIOFILM

As mentioned earlier, biofilms are an obstacle to the components of the host's immune system components. At present, traditional treatment methods for solving microbial infections, such as antibiotics, are mainly used. Because of the medical significance of bacterial biofilms, efficacious ways for biofilm are of great importance for clinical application. In fact, some potentially effective clinical interventions for the treatment of bacterial biofilms related to infection have recently been proposed.

**Figure 5:**
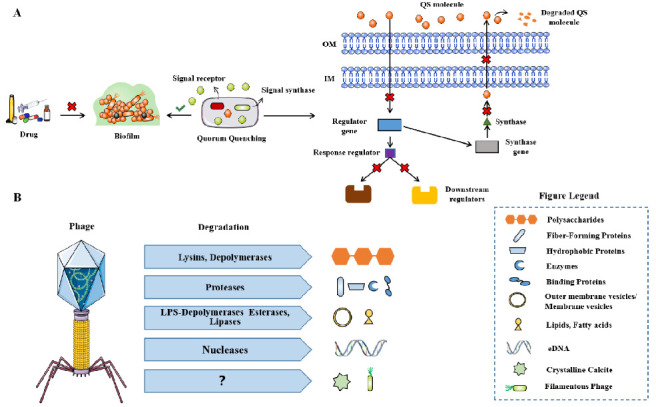
FIGURE 5: The mechanism of QQ for biofilm infection. **(A)** Phage-derived enzymes that degrade extracellular polymeric substances (EPS) of biofilms **(B)**. Modified from Azeredo [128].

QS is the most important regulatory pathway involved in biofilm formation that has been identified. Therefore, disrupting QS is known as Quorum quenching (QQ) and may be a promising method for treating biofilm related infections (**Fig. 5A**). QQ can occur at multiple levels, preventing bacterial adhesion, inhibiting biofilm maturation, or leading to mature biofilm decomposition. Although QQ cannot kill bacteria, it makes bacteria more susceptible to traditional treatments, for example, in combination with antibiotics. Several such methods have been reported to successfully treat *S. aureus* and *P. aeruginosa* biofilms [107]. In this review, it is pointed out that c-di-GMP is a significant signal molecule in many bacteria that promotes biofilm formation [108]. Inhibitors of diguanylate cyclase have recently been found and have been proven to effectively inhibit biofilm synthesis in *P. aeruginosa* and *Acinetobacter baumannii* [109].

The use of bacteriophages is considered to be a favorable alternative treatment for antibiotics [110]. The same strategy is also applicable to biofilm related infections, because some phages are known to have hydrolases on their surface, allowing them to invade biofilm matrix and infect bacteria in biofilm (**Fig. 5B**) [111]. The alginase secreted by *P. aeruginosa* specific phages, degrades the alginic acid capsule in cystic fibrosis patient as reported by Glonti T *et al.* [112]. In addition, lysogenic phages can not only provide a useful and multifunctional tool for inducing the lysis of biofilm root cells through genetic engineering, but also regulate their behavior through many other approaches [113]. Phage therapy has been shown to be effective in improving *P. aeruginosa* biofilm infection in patients with chronic otitis media and mouse chronic lung infection model [114, 115]. Phage therapy combined with previous biofilm debridement significantly improved the wound healing of the chronic *S. aureus* wound infection model [116]. Nevertheless, the use of phages still has limitations, such as the risk of bacterial resistance to phages, the possibility of unwanted horizontal gene transfer through lysogenic phages to share gene elements related to virulence in the whole biofilm community, and the immunogenicity leading to the production of neutralizing antibodies in human hosts, which may convert to inflammatory side effects [113].

Targeting matrix will destroy the stability of biofilm structure, which paves the way for the eradication of infection through the immune system, and improve the penetration of antibacterial molecules in biofilm. The method of dissolving biofilm matrix has been proved to be effective *in vitro* and animal experiments, but it has not been used in clinic [117–119]. A phase 2 clinical study (NCT03822455) using alginate oligosaccharide (Oligo G) with biofilm destruction properties in patients with cystic fibrosis is ongoing [118]. DNAse is commonly used to treat chronic pulmonary infection caused by *P. aeruginosa* in CF patients to destabilize eDNA, the main source of which is destroyed PMNs [120]. Alternatively, enzymes cause depolymerization of the matrix or activation of natural dispersal mechanisms [119,121,122]. Dispersin B is a glycoside hydrolase with the activity of dispersing biofilm and can inhibit biofilm formation of *S. epidermidis, E. coli* and *S. aureus* and disperses *E. coli* and *S. epidermidis* biofilms. Low dose nitric oxide administration was shown to stimulate *P. aeruginosa* biofilm dispersal through a decrease in c-di GMP levels [123]. Recently, small molecules that inhibit c-di GMP, able to disperse *P. aeruginosa* biofilms have been identified [124]. Like all agents stimulating biofilm dispersal, matrix degrading molecules would need to be combined with antibiotics to prevent bacteria from spreading to other parts of the body. Acquirement of right concentration of matrix dispersing agents and antibiotics at the site of infection might be a challenge for the translation of this strategy to the clinic [125].

Because the low metabolic state of bacteria is the common feature of tolerant bacterial biofilm population, regulating metabolic activity is a possible way to improve the efficacy of bactericidal antibiotics. However, these strategies have not been tried in biofilm growing bacteria, although they have been shown to play a role in fixed-phase cells, such as aminoglycosides combined with tricarboxylic acid cycle metabolites and ciprofloxacin combined with glucose and fumarate [126]. Several strategies against persistent cells have been proposed and divided into three categories: killing metabolic dormant persistent cells, bypassing the need for active cell processes, resuscitating persistent cells to be sensitive to antibiotics, and interfering with the formation of persistent cells [127]. *In vivo*, these strategies theoretically increase the risk of infection transmission, which may be a concern for immunocompromised patients who need active antibiotic treatment with bactericidal compounds at the same time.

## CONCLUSION

Bacterial biofilms are closely related to human health, because they involve various human diseases and show high tolerance and drug resistance. Consequently, it is urgent to study strategies for biofilm therapy intervention. In this review, we introduced the formation and characteristics of biofilm, and explored the general tolerance and drug resistance mechanism of biofilm infection. The increase in mutations and horizontal gene transfer are related to the rapid development of drug resistance in microbial biofilm. In addition, the formation of biofilm will promote the occurrence and development of many diseases. The complexity of biofilm needs to be considered in the diagnosis and treatment of many chronic diseases.

In the future, it is necessary to use multi-omics technology and bioinformatics technology to analyze the formation mechanism of various microbial biofilms and related QS mechanisms, develop natural QQ agents, and prevent the formation of biofilms from a biological perspective; multidisciplinary efforts are needed to develop interface materials to prevent biofilm colonization and strategies to effectively destroy biofilm matrix; It is necessary to adjust the pharmacokinetics and pharmacodynamics of biofilm infection treatment at this stage, so as to effectively remove biofilm infection and avoid collateral damage to surrounding tissues; At the same time, it is necessary to translate the *in vitro* results of effective therapeutic drugs into *in vivo* systematic clinical trials and develop a closer *in vivo* biofilm model.

## Abbreviations:

EPS: – extracellular polymeric substance,
eDNA: – extracellular DNA,
c-di-GMP: – cyclic diguanoside-5'-monophosphate,
PAMPs: – pathogen associated molecular patterns,
LPS: – lipopolysaccharides,
PMNs: – polymorphonuclear leukocytes,
PRRS: – pathogen recognition receptors,
BAMPs: – biofilm related molecular patterns,
Pel: – pellicle,
Psl: – polysaccharide synthesis locus,
HGT: – horizontal gene transfer,
TLS: – translational synthesis,
ARGs: – antimicrobial resistance genes,
QS: – quorum-sensing,
NIH: – National Institutes of Health,
CF: – cystic fibrosis,
COPD: – chronic obstructive pulmonary disease,
IBD: – inflammatory bowel disease,
QQ: – quorum quenching,
Oligo G: – alginate oligosaccharide.
